# Histone deacetylase 3 inhibition re-establishes synaptic tagging and capture in aging through the activation of nuclear factor kappa B

**DOI:** 10.1038/srep16616

**Published:** 2015-11-18

**Authors:** Mahima Sharma, Mahesh Shivarama Shetty, Thiruma Valavan Arumugam, Sreedharan Sajikumar

**Affiliations:** 1 Department of Physiology, Yong Loo Lin School of Medicine, National University of Singapore, Singapore-117 597; 2 Neurobiology/Aging Program, Life Sciences Institute (LSI), National University of Singapore, Singapore-117 456.

## Abstract

Aging is associated with impaired plasticity and memory. Altered epigenetic mechanisms are implicated in the impairment of memory with advanced aging. Histone deacetylase 3 (HDAC3) is an important negative regulator of memory. However, the role of HDAC3 in aged neural networks is not well established. Late long-term potentiation (late-LTP), a cellular correlate of memory and its associative mechanisms such as synaptic tagging and capture (STC) were studied in the CA1 area of hippocampal slices from 82–84 week old rats. Our findings demonstrate that aging is associated with deficits in the magnitude of LTP and impaired STC. Inhibition of HDAC3 augments the late-LTP and re-establishes STC. The augmentation of late-LTP and restoration of STC is mediated by the activation of nuclear factor kappa B (NFκB) pathway. We provide evidence for the promotion of associative plasticity in aged neural networks by HDAC3 inhibition and hence propose HDAC3 and NFκB as the possible therapeutic targets for treating age -related cognitive decline.

Aging is commonly associated with cognitive deficits^1^. The frequency of age-related cognitive decline is increasing dramatically as human life span increased over the last few decades. Performance in the tasks requiring associative information processing is also known to get impaired with aging^2^,^3^ mainly because of the vulnerability of the brain structures involved in it, such as hippocampus^4^,^5^. Synaptic plasticity such as long-term potentiation (LTP) and its associative mechanism such as synaptic tagging and capture (STC) are considered as the cellular basis of long-term memory^6^ and associative memory^7^,^8^. STC proposes the synaptic tag- plasticity related products (PRPs) interaction, where the tag is created by the weak stimulus or weak memory trace and PRPs are induced by strong stimulus or strong memory trace in two independent synaptic inputs of the same neuronal population^8^,^9^.

LTP is largely impaired in the aged rats at Schaffer collateral CA1 synapses^10^,^11^. The deficits in the late-LTP are correlated with age- related defects in memory^12^,^13^. Associative memory is also affected with aging but the molecular mechanisms are largely unknown^14^. The cognitive decline with aging is believed to be associated with aberrant changes in gene expression resulting from the dysregulated epigenetic mechanisms^15^,^16^. The epigenetic changes include DNA methylation and post translational modification of histones^15^. The most widely studied histone modification that is a critical regulator of memory formation is histone acetylation^17^. Histone acetyltransferases (HATs) and Histone deacetylases (HDACs) are the enzyme modifiers that function antagonistically to each other. Altered histone acetylation is associated with memory impairment in aged mice^18^. The HDAC inhibitors enhance LTP *in vitro* and augment memory formation *in vivo* in normal rodents and in a neurodegeneration model^18^,^19^,^20^,^21^. The broad spectrum HDAC inhibitors mostly affect Class I HDACs with little effect on Class II HDACs^22^. HDAC3 is the most highly expressed class I HDAC in the brain with greatest expression in the neurons of hippocampus, cortex, and cerebellum^23^ and is a critical negative regulator of learning and memory^24^,^25^. Selective inhibition of HDAC3 enhances the memory^26^. HDAC3 efficiently inhibits the nuclear factor κB (NFκB) activation by forming a corepressor complex (HDAC3/NCoR)^27^. NFκB, a transcription factor, is localized in both neurons and glia and plays an important role in the survival and plasticity of neurons^28^. During the induction of LTP, NFκB gets activated and induces the expression of genes such as *bdnf*, *Zif268*, *transthyretin* and *fos*, which are implicated in memory^29^,^30^.

In the present study, we investigated the possible involvement of HDAC3 in LTP and STC process in the aged hippocampal neural network since HDAC3 has higher expression in the hippocampus^23^,^24^. Inhibition of HDAC3 during the induction of late plasticity enhanced the magnitude of LTP and re-established associative plasticity in aged neural networks. We report that, with aging, HDAC3 prevents NFκB activation. Our findings highlight that the inhibition of HDAC3 in aged neural network is beneficial for re-establishing associative plasticity through the activation of NFκB pathway.

## Results

### HDAC3 inhibition re-establishes protein synthesis and NMDA receptor dependent late-plasticity

**Figure 1:**
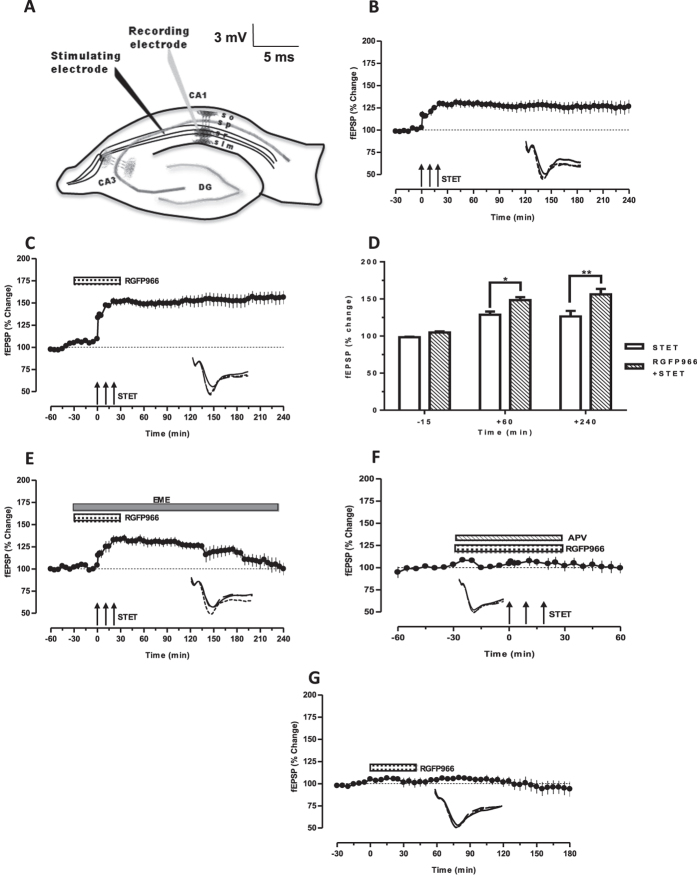
HDAC3 inhibition in aged neural networks augments LTP. (**A**) Schematic representation of a hippocampal slice depicting the location of electrodes in CA1 region for field-EPSP recording: Stimulating electrode was positioned in stratum radiatum to stimulate Schaeffer collateral and recording electrode was positioned onto CA1 apical dendrites. (**B**) The STET resulted in potentiation that maintained for 4 hours (n = 8). (**C**) The induction of LTP in the presence of HDAC3 inhibitor, RGFP966 (10 μM) resulted in augmented potentiation that lasted for 4 hours (n = 9). (**D**) A histogram of mean fEPSP slope values recorded for ‘STET’ groups and ‘RGFP966 + STET’ groups at the time points −15 minute (baseline), +60 minute and +240 minute analyzed with two-way ANOVA. Analysis between groups with Tukey’s multiple comparisons test showed significant differences between the ‘STET’ and ‘RGFP966 + STET’ groups at 60 minute (*P* < 0.05) and 240 minute (*P* < 0.001). (**E**) STET coupled with co-application of RGFP966 (10 μM) and Emetine (20 μM) resulted in a potentiation that decayed back to baseline (n = 6). (**F**) STET could not induce potentiation in the presence of APV (50 μM) and RGFP966 (10 μM) (n = 3). (**G**) The bath application of RGFP966 (10 μM) did not significantly alter the baseline responses (n = 6). Three solid arrows in all the figures represent the STET for the induction of late-LTP. Representative fEPSP traces shown in B to E were recorded at -15 min (solid line); 75 minute (dotted line) and 240 minute (hatched line) after LTP induction. In F, the post induction traces are from +30 and +60 min whereas in G it represents +75 and +180 min. Scale bars for all the traces vertical: 3 mV; horizontal: 5 ms. Asterisks indicate significant differences between groups (Tukey’s post hoc test, *P < 0.05, **P < 0.001, ***P < 0.0001). Error bars indicate ± SEM.

Previous studies have reported deficits in LTP with aging^2^,^31^. Curtailment of LTP persistence has been observed in aged rodents, while the induction was normal^12^,^13^. The first series of experiments involved the induction of late-LTP by high frequency stimulation at the CA1 Schaffer collateral synapses in the hippocampal slices of aged rats. The resulting potentiation was comparatively lesser than that observed in normal adult rats (5–7 week) (Supplementary Fig. 1B). The fEPSP slope potentiation became significantly different from the baseline after the tetanization (117.2 ± 4.348%; *P* *=* 0.0078) and maintained for the entire recording period of 4 hours. The mean potentiation at the end of 4 hours was 126.3 ± 7.555% (n = 8, Fig. 1B).

HDAC activity is reported to increase in the aged rat brain^32^. We investigated whether HDAC3 inhibition has any effect on the functional plasticity of synapses in aged neural networks, using a selective HDAC3 inhibitor, RGFP966^26^. After recording a stable baseline of 30 minutes, RGFP966 (10 μM) was bath applied to the slices for the next 60 minutes. Late-LTP was induced at the 30^th^ minute after the start of RGFP966 application (thus, a total of 60 minutes baseline). As shown in Fig. 1C, the induction and persistence of late-LTP was significantly increased as compared to the control late-LTP (Fig. 1B). Statistically significant potentiation was observed from 1 minute (133.2 ± 3.056% *P* = 0.0039) to 240 minutes (156.2 ± 7.472%, *P* = 0.0039, n = 9). A comparison of the potentiation of Fig. 1B, C is presented as bar graph (Fig. 1D).

We further tested the protein synthesis and NMDA receptor dependency of RGFP966-facilitated LTP in aged rats. Emetine (20 μM), a protein synthesis inhibitor, was co-applied with RGFP966 (10 μM) for 30 minutes and late-LTP was induced by STET. The bath application of RGFP966 was discontinued 30 minutes after STET while emetine application lasted till the end of experiment similar to that of the earlier report^33^. Statistically significant potentiation was observed up to 120 minutes (126.2 ± 3.959% *P* = 0.0313) and it gradually decreased and maintained at baseline potentials. The mean potentiation at the end of 4 hours was 99.93 ± 7.087% which was not significantly different from its baseline (Fig. 1E; *P* = 1.000, n = 6). To further assess that RGFP966-facilitated LTP is NMDAR-dependent, AP-5 (50 μM) was co-applied with RGFP966 for a period of 60 minutes after recording a stable baseline of 30 minutes. Late-LTP was induced 30 minutes after the drug application. No significant potentiation was observed in this case. The potentials remained at the baseline level throughout the recording (99.42 ± 5.745%, *P* > 0.05, n = 3; Fig. 1F). The results suggest that the augmentation of LTP by HDAC3 inhibition is protein synthesis and NMDA receptor dependent.

To test whether the bath application of RGFP966 alone has any long-term non-specific effects on the baseline potentials, RGFP966 (10 μM) was bath applied for 60 minutes after recording a stable baseline of 30 minutes. The mean potentiation at the end of 180 minutes (93.83 ± 8.444%) was not statistically significant compared to the pre-drug application baseline (97.58 ± 1.814%; *P*>0.05, n = 6, Fig. 1G).

### HDAC3 inhibition restores STC in aged neural networks

**Figure 2:**
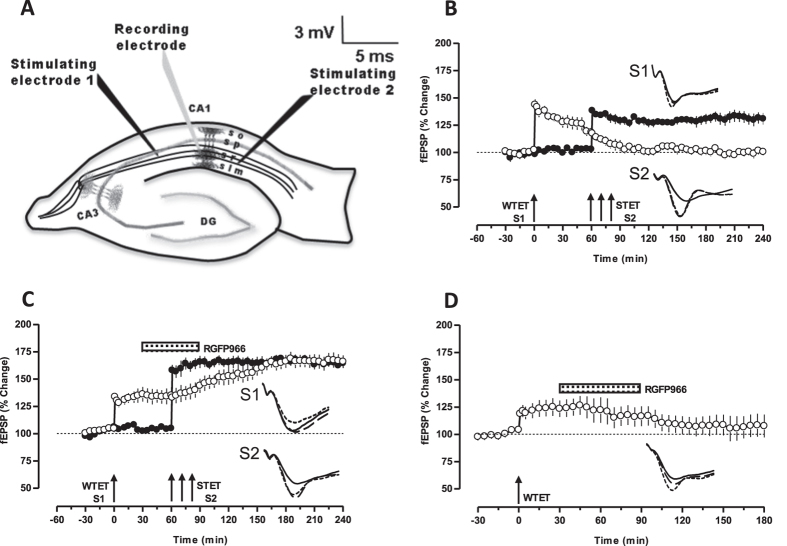
HDAC3 inhibition re-establishes synaptic tagging and capture. (**A**) Schematic representation of a hippocampal slice depicting the location of electrodes in CA1 region for the synaptic tagging and capture experiments. S1 and S2 are two stimulating electrodes positioned in the stratum radiatum to stimulate two independent synaptic inputs to the same neuronal population. The recording electrode is placed midway between S1 and S2 to record fEPSP from the distal apical dendrites. (**B**) “Weak before strong” paradigm was used to study STC. STET in S2 (filled circles) resulted in a significant potentiation that maintained till the end of recording but the WTET in S1 (open circles) resulted in early-LTP that was not reinforced (n = 6). (**C**) Inhibition of HDAC3 restored the synaptic tagging and capture. STET in the presence of RGFP966 (10 μM) induced a significant potentiation in S2 (filled circles) and the early-LTP in S1 (open circles) is transformed into a long-lasting LTP (n = 7). (**D**) RGFP966 (10 μM) applied 30 minutes after the WTET could not reinforce early-LTP to late-LTP. The potentiation was significant till 45 minutes (*P* < 0.05) but gradually declined to baseline (*P* > 0.05, n = 7). Single arrow represents the time of induction of eary-LTP by weak tetanization (WTET). All other symbols and traces are similar to Fig. 1 except in 2D, which is similar to Fig. 1G. Scale bars for all the traces vertical: 3mV; horizontal: 5ms. Error bars indicate ± SEM.

To test whether aged neural networks express associative plasticity, we conducted STC experiments using a two pathway model (Fig. 2A). The “weak before strong” paradigm^34^ was used to induce early-LTP in synaptic input S1 and late-LTP in S2 within an interval of 60 min. This time period was shown to express STC in adult slices (Supplementary Fig. 2). Interestingly, the potentiation in S1 was significant up to 30 minutes (127.7 ± 4.988%, *P* = 0.0313) after which it gradually decayed to baseline with the mean potentiation of 100.5 ± 1.373% at the end of 4 hours that was not statistically significant different from its baseline (Fig. 2B, open circles *P* = 0.8438). The potentiation in S2 became statistically significant after the induction (146.7 ± 8.718%, *P* = 0.0313) and it maintained till the end of recording (141.0 ± 11.10%, filled circles, n = 6). Since the inhibition of HDAC3 augmented late-LTP; we asked whether the inhibition of HDAC3 can restore STC. We tested this hypothesis by studying STC in the presence of HDAC3 inhibitor. After a stable baseline of 30 minutes, early-LTP was induced in input S1. RGFP966 was bath applied 30 minutes after the induction of early-LTP in S1 and continued for the next 60 min. Late-LTP was induced in S2 at 60^th^ minute in presence of the drug. Unlike the control experiment (Fig. 2B), early-LTP in S1 transformed to late-LTP, expressing STC. The potentiation in both S1 (165.8 ± 5.820%, *P* = 0.0156) and S2 (164.4 ± 2.552%, *P* = 0.0156) was statistically significant for the entire period of 240 minutes (n = 7, Fig. 2C).

To rule out the possibility that HDAC3 inhibition after the induction of early-LTP results in late-LTP, we conducted a control experiment in which the inhibitor was applied 30 minutes after WTET in S1 and the drug application continued for next 1h, a similar time frame as of Fig. 2C. We could not observe reinforcement of early-LTP (Fig. 2D). The potentiation became significantly different from the baseline value following the induction (118.8 ± 5.037%, *P* = 0.0156) till 45 minutes (126.9 ± 7.473%, *P* = 0.0313) after which it gradually declined, depicting a normal early-LTP with the mean potentiation of 107.6 ± 10.54% at the end of 3 hours which was similar to that of baseline (Fig. 2D, *P* = 0.0781, n = 7).

### HDAC3 inhibition enhances STC by activating NFκB pathway

**Figure 3:**
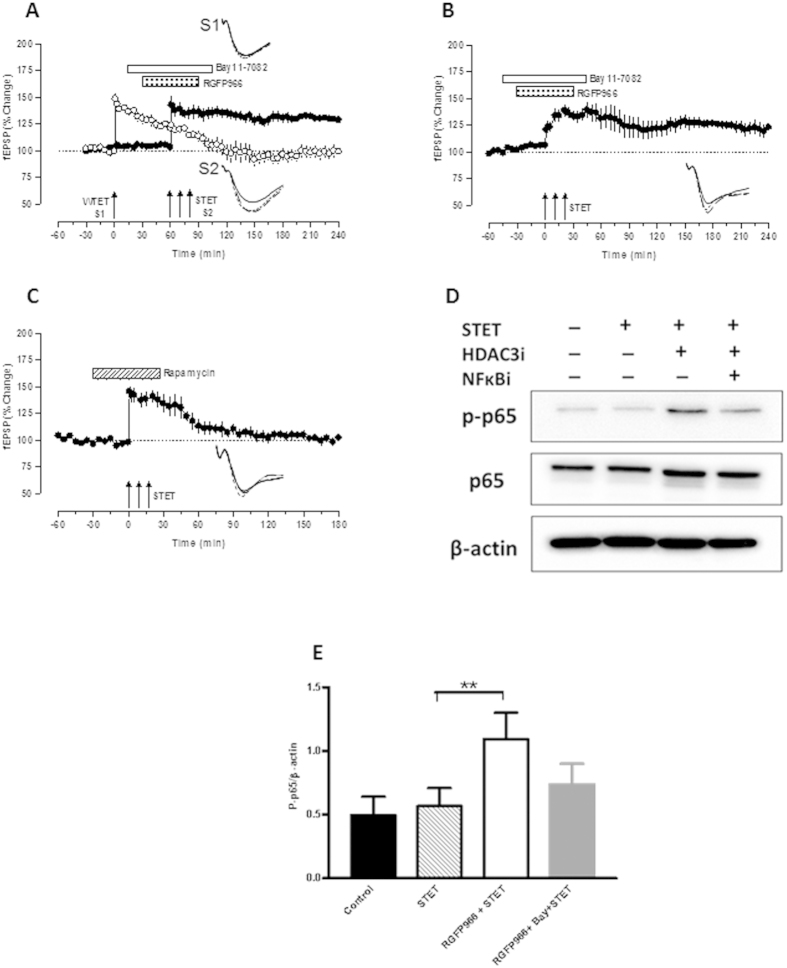
HDAC3 inhibition restores synaptic tagging and capture through the activation of Nuclear factor κB. (**A**) Inhibition of HDAC3 failed to restore associativity in the aged neural networks when the activation of NFκB is prevented. WTET in S1 (open circles) after recording a stable baseline of 30 minutes resulted in potentiation significant up to 85 minutes after which it gradually decayed to baseline. STET in the presence of RGFP966 (10 μM) and Bay 11-7082 (30 μM) resulted in a significant potentiation in S2 (filled circles) that maintained for 4 hours (n = 6). Thus, STC was not expressed. (**B**) STET in the presence of Bay 11-7082 (30 μM) and RGFP966 (10 μM) induced a significant potentiation that lasted for 4 hours (n = 6). (**C**) STET in the presence of rapamycin (0.1 μM) resulted in potentiation that was significant up to 50 minutes. Afterwards, the potentiation rapidly decayed to baseline (n = 6). (**D**) Western blot analysis of hippocampal slices revealed increased levels of phospho-p65 in the ‘RGFP966 + STET’ group (Group iii) in comparison to ‘control’ (Group i), ‘STET’ group (Group ii) and ‘RGFP966 + Bay11-7082 + STET’ group (Group iv). (**E**) Histogram showing differences in the relative amount of phospho-p65 in control (Group i), ‘STET’ (Group ii), ‘RGFP966 + STET’ (Group iii) and ‘RGFP966 + Bay11-7082 + STET’ (Group iv) (one-way ANOVA, F = 17.33; *P* < 0.01). The values of the individual groups were calculated in relation to the control group while β-actin serves as a loading control. Each bar represents mean ± SEM of analysis of 5 blots per group. Asterisk indicates significant difference (Dunnett’s *post hoc* test, **P < 0.01). Symbols and traces are similar to Figs 1 and 2 except Fig. 3C which is similar to Fig. 1G and 2D.

Having observed the facilitation of LTP and restoration of associative plasticity by HDAC3 inhibition, we were interested to know about the molecular mechanisms underlying the augmentation of late-LTP by HDAC3 inhibition and the possible molecular players that maintain STC during the inhibition of HDAC3. HDAC3 has been reported to efficiently inhibit NFκB activation^35^ which is further known to regulate synaptic plasticity^28^,^29^,^30^. To assess whether the effects of HDAC3 inhibition on late-LTP and STC in aged rats were NFκB activation dependent, we performed a series of experiments using a NFκB inhibitor, Bay11-7082^36^. Bay11-7082 is a selective and irreversible inhibitor of NFκB activation that blocks activity dependent phosphorylation of inhibitor of κB α (IκBα) without affecting constitutive IκBα phosphorylation. To investigate whether HDAC3 inhibition restores the STC via NFκB, WTET was applied in S1 after recording a stable baseline of 30 minutes. Fifteen minutes later, Bay11-7082 (30 μM) was bath applied for duration of 15 minutes followed by co-application of Bay11-7082 and RGFP966 for the next 60 minutes. Late-LTP was induced in S2, 45 minutes after the application of Bay11-7082. The total duration of Bay11-7082 application was 90 minutes. The potentiation in S2 was statistically significant throughout the recording with a mean potentiation of 128.7 ± 1.586% at the end of 4 hours (Fig. 3A, *P* = 0.0313). However, the potentiation in S1 was significantly different from the baseline till 85 minutes (115.0 ± 3.240%, *P* = 0.0313) after which it gradually declined to the baseline. The mean potentiation at the end of 4 hours in S1 was 98.90 ± 5.323% which was not significantly different from its baseline (Fig. 3A, open circles, *P* = 1.000, n = 6). These data provide evidence that NFκB is a plasticity factor that is activated during the inhibition of HDAC3, which further maintains the STC in aged neurons.

In the subsequent experiment, Bay11-7082 was bath applied to the slices for 15 minutes followed by the co-application of Bay11-7082 and RGFP966 (10 μM) for 60 minutes. Bay11-7082 application was continued for another 15 minutes. STET was delivered 45 minutes after the start of Bay11-7082 application (thus the baseline was for a period of 75 minutes). As shown in Fig. 3B, the potentiation towards the end of recording was similar to that of normal aged rat. Statistically significant potentiation was observed from 1 minute (133.7 ± 4.719%) to 240 minutes (122.9 ± 4.201%, *P* = 0.0313, n = 6). Thus, in this case, late-LTP was maintained but there was no augmentation of LTP as observed in case of HDAC3 inhibition. The result suggests that HDAC3 inhibition indeed leads to the activation of PRPs, possibly through the activation of NFκB pathway, which is critical for the observed augmentation of LTP.

Next we checked the possible reason for the maintenance of late-LTP during NFκB inhibition as seen in Fig. 3A. Rapamycin (0.1 μM), mammalian target of rapamycin (mTOR) inhibitor, was bath applied for duration of 60 minutes after recording a stable baseline of 30 minutes. Late-LTP was induced in presence of rapamycin and potentiation in this case was significant only up to 50 min (Fig. 3C, 121.8 ± 3.576%, *P* = 0.0313) after which it rapidly decayed to baseline. The mean potentiation at the end of 3 hours (102.6 ± 1.612%) was similar to its baseline (*P* = 0.1563, n = 6). The result suggests that this form of LTP is maintained by mTOR-mediated protein synthesis.

To support the above electrophysiology observations that HDAC3 inhibition restores STC via activation of NFκB, we quantified the levels of phospho-p65 (a marker of NFκB activation). Immunoblot analysis revealed a significant increase in the phospho-p65 levels following the induction of LTP in ‘RGFP966 + STET’ group compared with either ‘control group’ or ‘STET’ group or ‘RGFP966+ Bay + STET’ group (Fig. 3D). A one-way analysis of variance (ANOVA) comparing the normalized levels of phospho-p65 to control (0.5) showed a significant difference between the means (F = 17.33; *P* = 0.0077). A multiple comparison with Dunnett’s *post hoc* test showed that the increase in the amount of phospho-p65 in ‘RGFP966 + STET’ group was statistically significant (*P* = 0.0037, n = 5) as compared to control group and ‘STET’ group (Fig. 3E). Thus, HDAC3 inhibition results in the activation of NFκB, which enhances late-associativity in the aged neural networks.

## Discussion

Age associated memory impairment has been reported to be correlated with deficits in LTP^12^,^13^,^37^. The decrease in the magnitude of LTP observed in our study is consistent with the earlier reports^2^,^11^,^31^. It has been reported earlier that the hippocampal granule cells or CA1 pyramidal cells in old rats preserve most of the biophysical properties and spontaneous firing rates^38^,^39^. In CA1 pyramidal neurons, the field EPSP elicited by Schaffer collateral stimulation was reduced without changing the presynaptic-fibre potential response^40^,^41^ and in addition a reduction in the actual number of perforant-path synaptic contacts on granule cells were observed^42^, contributing to decreased network connectivity and plasticity changes with advanced aging^43^,^44^. Our finding are in agreement with the recent report that with aging, dysregulation of synaptic protein synthesis occurs that contributes to the age-dependent reduction in LTP persistence. Another possible reason for the impaired plasticity could be the alterations in calcium-mediated cascades as an increased density of L-type calcium channels in old rats has been reported^45^.

Associativity is one of the basic properties of functional neural networks and is governed by different neuromodulators such as dopamine and the amount of proteins synthesized during induction of a plastic event^8^,^46^. Age dependent decrease in protein synthesis is known to affect memory formation^47^ and possibly late- association such as STC. Indeed, we found that the STC is impaired in the aged rats. It reveals an interesting aspect of aged neural networks that those networks are able to create and maintain plasticity but are unable to associate weak and strong synapses to form long-term associative plasticity. These results are consistent with the recent findings from humans that lack of dopaminergic modulation with aging leads to impairment in associative memory^48^. Our findings provide evidence that mTOR mediated protein synthesis is capable of maintaining late-LTP in aged neural networks but it is unable to drive the synapses for late-association such as STC. These results are in agreement with our earlier report that certain types of stimulation are able to create plasticity but fail to initiate and maintain associativity^49^. The possible reason for the lack of late-associativity in aged neural networks could be due to the lack of a pool of plasticity products during the induction of long-term plasticity. We have reported earlier that late-associative interactions such as STC and cross-capture utilize plasticity proteins from a common pool^50^. The pool of plasticity products includes, calcium-calmodulin kinase II (CaMKII), calcium-calmodulin kinase IV (CaMKIV), mitogen activated protein (MAP) kinases, brain derived neurotrophic factor (BDNF) and protein kinase Mζ (PKMζ), which are captured by potentiated or depressed synapses^50^.

HDAC3 is the most highly expressed Class I HDAC in the brain. HDAC activity increases with aging^32^. Wood and colleagues reported that the selective inhibition of HDAC3 can enhance memory processes involved in extinction of drug-seeking behavior^25^,^26^. We provide further evidence that inhibition of HDAC3 in aged neural networks is beneficial in promoting synapses for coding memory through association on a long-term basis, which is of critical importance because associative memories are largely impaired in neurodegenerative diseases such as Alzheimer’s disease (AD).

It has been reported earlier that HDAC3 efficiently inhibits NFκB dependent gene transcription^35^,^51^. NFκB has been shown to facilitate the synaptic activity dependent *de novo* gene transcription and is critical for long-term memory formation^52^. In addition, another study has highlighted the important role of NFκB in cognitive functions such as inhibitory avoidance long-term memory^53^. Interestingly, we found NFκB -mediated mechanisms to be critical in the augmentation of LTP and re-establishment of STC observed with HDAC3 inhibition. This is also supported by our findings showing increased level of phospho-p65, a marker of active NFκB, with the inhibition of HDAC3. Our findings are consistent with a recent DNA microarray analysis study by Williams and colleagues, in which they compared LTP-associated gene expression in young, middle-aged, and old male rats. The authors found that the overall expression of plasticity genes in young group is highly regulated but noticed dysregulation of activator protein-1 and NFκB transcription factor activity.

**Figure 4:**
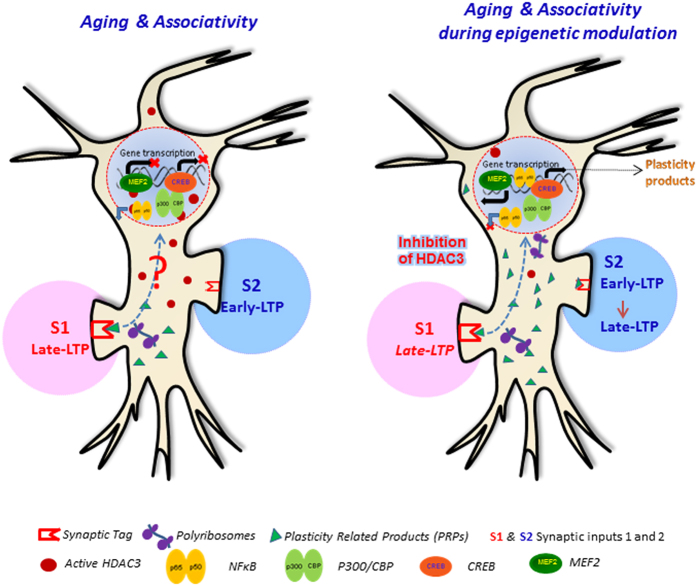
Schematic model for the rescue of associative plasticity in aged neural networks by inhibition of HDAC3. (**A**) In aged neural networks, induction of late-LTP by STET marks synapses (S1) with a synaptic ‘tag’ and induces protein synthesis. The subsequent induction of E-LTP in the neighbouring synapses (S2) by WTET leads to synaptic competition for the available plasticity realted products (PRPs). Here, the synapse S1 completely utilizes the PRPs for its own maintenance without sharing it with the other synapses in S2. Thus, there is no late-associativity between these synapses. Increased activity of HDAC3 is one of the possible contributing factors. The active HDAC3 deacetylates its histone and non-histone (NFκB, CREB binding protein CBP, myocyte enhancer factor 2 MEF2) targets. The active HDAC3 prevents the nuclear gene transcription by promoting the export of NFκB from the nucleus; decreasing the CBP histone acetyltransferase (HAT) activity; and by terminating the MEF2-dependent transcription of plasticity genes. (**B**) The inhibition of HDAC3 activity prevents the export of NFκB from the nucleus. As a result, NFκB signaling cascade is activated that results in the induction of numerous gene targets, many of which code for the plasticity proteins such as BDNF and CaMKII. In addition to this, CREB-mediated gene transcription and MEF2-dependent transcription of structural plasticity genes is also enhanced. Subsequently, the PRPs get globally distributed in the neuron and are shared between both the active synapses (S1 and S2) resulting in a stable plasticity and late-associativity (adapted and modified from Chen et., 2001; McQuown and Wood 2011).

HDAC3 can deacetylate the p65 component of NFκB and promote its export from the nucleus^51^. As a result, NFκB might not be available for binding to the κB enhancer element of its target genes and induce their transcription. HDAC3 can affect plasticity and late associativity by decreasing the CREB binding protein (CBP) activity or by terminating the myocyte enhancer factor 2 (MEF2) dependent transcription of structural plasticity genes (Fig. 4)^54^. Given the evidence that NFκB, CBP and MEF2 are all targets of HDAC3 and NFκB interacts with CBP/p300, an interesting possibility is the concerted regulation of activity of these three molecules by HDAC3 during aging. HDAC3 inhibition also leads to the increased expression of *Nr4a2, a* CREB-dependent gene implicated in memory^24^. Protein kinase A (PKA) and cAMP response element-binding protein (CREB), are identified as the downstream targets of active NFκB^55^,^56^. The other known targets are brain-derived neurotrophic factor (BDNF) and calcium- calmodulin kinase II δ (CaMKII δ)^56^. It is possible that NFκB might be inducing the synthesis of BDNF, an important PRP^8^,^49^,^57^. We and others have reported earlier that BDNF and its receptor tropomyosin receptor kinase B (TrkB) plays an important role in establishing associative plasticity by acting either as a PRP or a synaptic tag^49^,^58^. It can be speculated that BDNF might be restoring the late-associativity in aged rats by activating its downstream signaling pathways.

In summary, our results highlight the role of HDAC3 activity in the age related cognitive deficits. Our study shows that aging significantly affects LTP and presents the first evidence of the impaired associative information processing in the aged hippocampal CA1 neurons. Inhibiting the HDAC3 activity restores the associativity in the aged neural networks (Fig. 4A,B). Here, we report that the re-establishment of STC is brought about by the activation of NFκB-mediated signaling pathways.

## Materials and Methods

### Electrophysiology

A total of 70 hippocampal slices from 17 male Wistar rats (82–84 week old) and 14 slices from 7 (5–7 week old) male Wistar rats were used for electrophysiological recordings. Animals were maintained on a 12/12 hour light dark cycle, with food and water available *ad libitum*. All animal procedures were carried out in accordance with protocols R13-4656(A)13 and R13-5711(A1)14 approved by the Institutional Animal Care and Use Committees (IACUC) at the National University of Singapore. The rats were decapitated after anesthetization using CO_2_ and the brains were quickly removed into cold (2–4 °C) artificial cerebrospinal fluid (ACSF). The ACSF contained the following (in millimolars): 124 NaCl, 3.7 KCl, 1.0 MgSO_4_.7H_2_O, 2.5 CaCl_2_.2H_2_O, 1.2 KH_2_PO_4_, 24.6 NaHCO_3_, and 10 D-glucose, equilibrated with 95% O_2_–5% CO_2_ (carbogen; total consumption 16L/h). From each rat, 8–10 transverse hippocampal slices (400 μm-thick) were prepared from the right hippocampus by using a manual tissue chopper. The slices were incubated at 32 °C in an interface chamber (Scientific System Design) at an ACSF flow rate of 1 mL/minute. Since the number of aged animals was limited, a total of four interphase chambers were used simultaneously to conduct four different experiments from one rat.

In one-pathway experiments, one monopolar stainless steel electrode (5 MΩ; A-M Systems) was positioned within the stratum radiatum of the CA1 region for stimulating the synaptic input-1 (S1) (Fig. 1A). In two-pathway experiments, two electrodes (5 MΩ; A-M Systems) were positioned within the stratum radiatum of the CA1 region for stimulating two separate independent synaptic inputs S1 and S2, to one neuronal population (Fig. 2A). Pathway independence between S1 and S2 was tested by using standard paired-pulse stimulation protocol with an interpulse interval of 30 milliseconds as described previously^59^. One electrode (5MΩ; A-M Systems) was placed in the CA1 apical dendritic layer for recording the field EPSP (fEPSP: measured as its slope function), and signals were amplified by a differential amplifier (Model 1700, AM Systems) and digitized using a CED 1401 analog-to-digital converter (Cambridge Electronic Design). After the pre-incubation period of 3 hours, an input-output curve (afferent stimulation vs fEPSP slope) was plotted prior to experiments. To set the test stimulus intensity, a fEPSP of 40% of its maximal amplitude was determined. Biphasic constant current pulses were used for stimulation. Late-LTP was induced using three stimulus trains of 100 pulses (“strong” tetanus [STET], 100 Hz; duration, 0.2 msec/polarity; inter-train interval, 10 minutes). A weak tetanization protocol consisting of one 100 Hz train (21 biphasic constant-current pulses; pulse duration per half-wave, 0.2 ms)^57^ was used to induce early-LTP. The slopes of the fEPSPs were monitored online. The baseline was recorded for 30 minutes. For baseline recording and testing at each time point, four 0.2-Hz biphasic constant-current pulses (0.1 ms/polarity) were used^57^.

### Pharmacology

The HDAC3 inhibitor, RGFP966 (Abcam) and NFκB inhibitor, Bay 11-7082 (Cayman Chemical) were dissolved in DMSO as stock solutions (10 mM) and stored at −20 °C. Emetine dihydrochloride hydrate (Sigma), D-2-Aminuteso-phosphopentanoic acid (AP-5) (Tocris) and Rapamycin were prepared as concentrated stock solutions in DMSO, and were diluted in ACSF to obtain a final concentration of 0.1 μM, 20 μM and 50 μM respectively. The stocks were stored for not more than a week. Right before application, the stocks were diluted to the final concentration in ACSF, bubbled with carbogen and bath applied for specified durations. The drugs were protected from light during storage and bath application was carried out under dark conditions. The final DMSO concentration was kept below 0.1%, a concentration which has been shown to not affect basal synaptic responses^60^.

### Immunoblot analysis of phospho-p65 NFκB

For immunoblot analysis, we used four groups of slices and each of the group contained a sample size of 6–9 slices: (i) ‘control’ slices which were incubated in the interphase chamber for 3 h; (ii) ‘STET’ group; (iii) ‘RGFP966 + STET’ group; and (iv) ‘RGFP966 + Bay + STET’ group. In each group, the slices were collected after the corresponding electrophysiological recordings. Tissues around the recording electrodes were cut carefully and snap frozen in liquid nitrogen and stored in -80ºC. The tissues were then lysed in Tissue Protein Extraction reagent (T-PER; Thermo Scientific) containing protease inhibitor and phosphatase inhibitor (1:50 ratio) and lysed. After centrifugation for 35 minutes at 14500 × g, the protein concentration of the supernatant was determined using bicinchoninic acid (BCA) protein assay. 20 μg of total protein were subjected to SDS-PAGE and subsequent immunoblotting with antibodies against total p65 and phospho-p65 NFκB (1:1000; Cell Signaling Technology) and β-actin (1:10000; Sigma-Aldrich). The amount of phospho-p65 NFκB was quantified by densitometric measurement of western blots using ImageJ (NIH software). The densitometric values of each blot were normalized to the amounts of β-actin which served as loading control and were calculated in relation to the control group. The values of each data points were represented as mean of five independent experiments.

### Statistical analysis

The data were represented as mean ± SEM. The average percentage values of fEPSP slope per time point were subjected to statistical analysis with GraphPad Prism 6.0. Non-parametric tests were used for the data that did not conform to Gaussian distribution. Wilcoxon matched-pairs signed rank test was used to analyze the average values of the slope function of the fEPSP (mV/ms) per time point when compared within the group^49^. Multiple, between group comparisons for specified time points were performed with one-way or two-way ANOVA with Tukey’s *post hoc* test. Statistical significance was assumed at *P* < 0.05 (*P < 0.05 **P < 0.001 ***P *<* 0.0001). For the analysis of western blot results, one-way ANOVA with Dunnett’s *post hoc* tests at the *P* < 0.05 significance level was used.

## Additional Information

**How to cite this article**: Sharma, M. *et al.* Histone deacetylase 3 inhibition re-establishes synaptic tagging and capture in aging through the activation of nuclear factor kappa B. *Sci. Rep.* **5**, 16616; doi: 10.1038/srep16616 (2015).

## Supplementary Material

Supplementary Information
