# Supplementary material for: Histone deacetylase 3 inhibition re-establishes synaptic tagging and capture in aging through the activation of nuclear factor kappa B
Source: Sci Rep. 2015 Nov 18;5:16616. doi: 10.1038/srep16616 (PMC4649608; doi:10.1038/srep16616)
Supplement: Supplementary Information [file srep16616-s1.pdf]

Supplementary Documents

Histone deacetylase 3 inhibition re-establishes synaptic tagging and capture in aging through the activation of nuclear factor kappa B

Mahima Sharma[1, 2], Mahesh Shivarama Shetty[1, 2], Thiruma Valavan Arumugam[1] & Sreedharan Sajikumar[1, 2]*

[1] Department of Physiology, Yong Loo Lin School of Medicine, National University of Singapore, Singapore-117 597

[2] Neurobiology/Aging Program, Life Sciences Institute (LSI), National University of Singapore, Singapore-117 456

Legends for Supplementary Figures

Supplementary Figure.1

A) Schematic representation of a hippocampal slice with two stimulating (S1 and S2) and one recording electrodes to study late-LTP and synaptic tagging and capture experiments (STC). B) Late-LTP recorded from the hippocampal slices from 5-7 weeks old male wistar rats. After a stable baseline of 1 h in S1 (filled circles) and S2 (open circles), late-LTP was induced by STET in S1 (100 Hz 100 pulses with 10 min interval) which resulted in 175% potentiation lasting 4h (n=7). Control potentials from S2 remained stable for the entire recroding period.

Supplementary Figure.2

STC experiments using weak before strong paradigm. After a stable baseline of 1h in S1 and S2, WTET was applied in S1 (filled circles). Late-LTP was induced in S2 (open circles) 60 min after the WTET in S1 (filled circles). Here the early-LTP in S1 was transformed to late-LTP expressing STC (n=7)

WTET: Weak tetanization , STET: Strong tetanization. Single arrow represents the time point of induction of early-LTP by WTET. Triple arrow represents the time point of induction of late-LTP by STET.

A

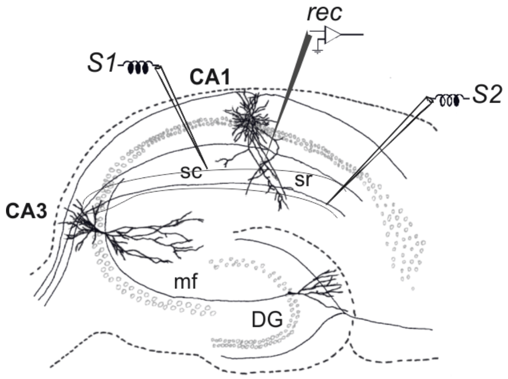

B

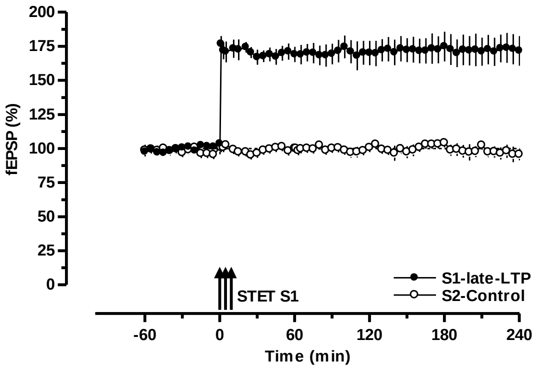

Supplementary. Figure.1

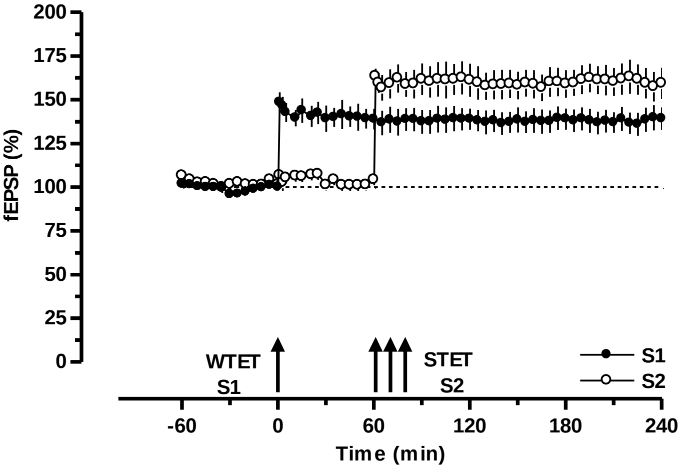

Supplementary. Figure.2
